# Farnesylation or geranylgeranylation? Efficient assays for testing protein prenylation *in vitro *and *in vivo*

**DOI:** 10.1186/1471-2091-7-6

**Published:** 2006-02-28

**Authors:** Wolfgang Benetka, Manfred Koranda, Sebastian Maurer-Stroh, Fritz Pittner, Frank Eisenhaber

**Affiliations:** 1 Research Institute of Molecular Pathology (IMP), Dr. Bohr-Gasse 7, A-1030 Vienna, Austria; 2 VIB – SWITCH lab, Pleinlaan 2, B-1050 Brussels, Belgium; 3 University Vienna, Department of Biochemistry, Dr.-Bohr-Gasse 9, A-1030 Vienna, Austria

## Abstract

**Background:**

Available *in vitro *and *in vivo *methods for verifying protein substrates for posttranslational modifications via farnesylation or geranylgeranylation (for example, autoradiography with ^3^H-labeled anchor precursors) are time consuming (weeks/months), laborious and suffer from low sensitivity.

**Results:**

We describe a new technique for detecting prenyl anchors in N-terminally glutathione S-transferase (GST)-labeled constructs of target proteins expressed *in vitro *in rabbit reticulocyte lysate and incubated with ^3^H-labeled anchor precursors. Alternatively, hemagglutinin (HA)-labeled constructs expressed *in vivo *(in cell culture) can be used. For registration of the radioactive marker, we propose to use a thin layer chromatography (TLC) analyzer. As a control, the protein yield is tested by Western blotting with anti-GST- (or anti-HA-) antibodies on the same membrane that has been previously used for TLC-scanning. These protocols have been tested with Rap2A, v-Ki-Ras2 and RhoA (variant RhoA63L) including the necessary controls. We show directly that RasD2 is a farnesylation target.

**Conclusion:**

Savings in time for experimentation and the higher sensitivity for detecting ^3^H-labeled lipid anchors recommend the TLC-scanning method with purified GST- (or HA-) tagged target proteins as the method of choice for analyzing their prenylation capabilities *in vitro *and *in vivo *and, possibly, also for studying the myristoyl and palmitoyl posttranslational modifications.

## Background

Prenylation is a lipid posttranslational modification (PTM) of proteins at cysteine residues in the C-terminal region [1-7]. The specific sequence environment recognized by prenyltransferases consists either of the CaaX box for farnesyltransferase (FTase) and geranylgeranyltransferase 1 (GGTase1) or C-terminal cysteines of Rab GTPases in the case of geranylgeranyltransferase 2 (GGTase2). In all instances, the cysteine-containing region must be preceded on the N-terminal side by approximately 10 residues providing a generally polar, flexible, so called linker segment without inherent conformational preferences [7]. The anchor can be of farnesyl (3 isoprenyl units) or of geranylgeranyl (4 isoprenyl units) type [8]. Targeting to cellular membranes [1,9] and mediation of protein-protein interactions [10-16] are well documented biological functions associated with these lipid anchors.

Members of the Ras family of GTPases are of particular medical interest, as their mutational hyperactivation as well as mutations of proteins lying upstream in their signaling pathways are associated with various cancers [17-24]. Several other CaaX proteins from the Rho family of GTPases [25,26] and Rap1A [27] are involved in tumorigenesis as well. Since their lipid modifications are essential for their biological function [10,28-31], inhibitors of prenyltransferases (PTases), especially of FTase [32-34] attracted the interest of pharmaceutical research as anti-cancer drugs. Two such compounds made it to phase III trials [35,36]. Furthermore, there is evidence that inhibitors of prenylation may be useful in the treatment of other diseases such as infestation with protozoa [6,37].

However, we are far from understanding the physiological consequences of inhibiting FTase or GGTase1 in cells since the lists of the respective substrates are essentially not known. Only a few dozen proteins, including several fungal lipopeptide pheromons [38,39] (e.g. a-mating factor of *Saccharomyces cerevisiae *[40,41]) as well as mammalian proteins of the Ras superfamily of small GTPases [42], the trimeric G proteins [43] and the nuclear lamins of type A [44] and B [45], have been experimentally identified and verified as substrates of specific prenyltransferases yet. Given the critical role of the prenyl anchor for biological function (both with respect to the occurrence of prenylation and to the dependence on anchor type), it is of growing interest to analyze the prenylation status of so far uninvestigated proteins and to enlarge the list of proven prenylated proteins. A recently developed sophisticated *in silico *method [46] generates a high number of predicted protein candidates for prenylation and, especially for twilight zone predictions, efficient methods for experimental verification of prenylation are necessary.

The standard literature method for *in vitro *or *in vivo *analysis of selected candidates involves transcription/translation of a cloned construct and protein prenylation in the presence of ^3^H-labeled lipid anchor precursors followed by autoradiography/fluorography [47-49]. Necessary controls involve mutations of the C-terminal cysteine expected to be modified, prenyltransferase inhibitor applications and/or exposition to precursors of alternative prenyl anchors during the prenylation reaction. However, the reportedly long exposure times (weeks/months) contradict the need for several repetitions of the experiment. Optimization of protein expression and incubation conditions is typically not avoidable. In our own experience, many attempts with the standard technology ended up without reportable result; i.e., the signals in initial experiments were often below the detection limit. Scientific literature research showed that rarely a lab has studied the prenylation status of more than a single target, apparently as a consequence of the tenacious methodology.

The problem of long exposure times for ^3^H-autoradiography has prompted us to study a variety of chromatographic and scintillation methods for developing a faster and more sensitive test system. We found a solution using a TLC linear analyzer for testing the prenylation of selected protein targets. N-terminally GST-tagged proteins were *in vitro *transcribed/translated and incubated with ^3^H-labeled anchor precursors. Such a quick *in vitro *screen might also be useful for finding proteins that deserve the effort for detailed *in vivo *studies. A similar approach can be used *in vivo*, if HA-tagged target proteins are expressed in cell culture supplemented by radioactive prenyl anchor precursors. This new approach on the detection of weak ^3^H-signals is expected also to be helpful for monitoring posttranslational modifications with similar ^3^H-labeled anchors such as myristoyl or palmitoyl.

## Results

### Optimization of experimental parameters and analysis of the prenylation behaviour of the protein Rap2A

The proposed new procedure starts with a PCR-amplification of the GST-Rap2A open reading frame (Genbank accession of Rap2A BC070031) followed by *in vitro *transcription and translation using rabbit reticulocyte lysate in the presence of a ^3^H-labeled isoprenoid donor. The GST-tagged target protein is purified utilizing glutathione sepharose 4B beads and concentrated by precipitation with acetone. The sample is subjected to SDS-page gel electrophoresis and transferred to a nitrocellulose membrane by electroblotting. Detection of incorporated radioactive label is performed by scanning with the TLC analyzer (scanning time: 20 minutes per lane). Afterwards, the amount of target protein is evaluated by Western blotting with an anti-GST-antibody on the same membrane.

Experiments with wildtype GST-Rap2A fusion protein and [^3^H]mevalonic acid were performed using various reaction times and amounts of radioactive label. The optimal conditions we found were 20–40 μCi [^3^H]mevalonic acid and at least four hours reaction time, which is in agreement with previous studies [48].

**Figure 1:**
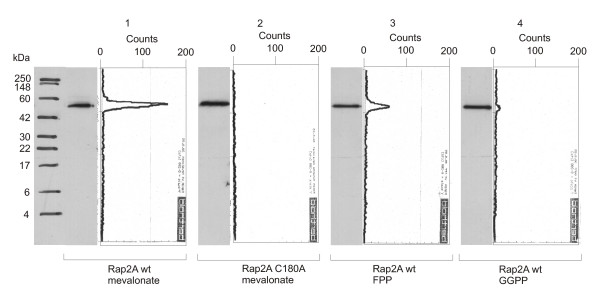
***Western blots and TLC scanning results for Rap2A with radioactive prenyl anchor precursors***. Western Blot and corresponding scans from TLC linear analyzer of wildtype GST-Rap2A-fusion protein translated with [^3^H]mevalonic acid (lane 1), GST-Rap2A C180A with [^3^H]mevalonic acid (lane 2), GST-Rap2A with [^3^H]FPP (lane 3) and GST-Rap2A with [^3^H]GGPP (lane 4). There is significant incorporation of a product of mevalonic acid (lane 1) as well as FPP (lane 3), while incorporation of GGPP is close to the detection limit (lane 4), suggesting that Rap2A is primarily a farnesylation target.

Experiments with 20 μCi [^3^H]mevalonic acid, 10 μCi [^3^H]farnesylpyrophosphate (FPP) and 10 μCi [^3^H]geranylgeranylpyrophosphate (GGPP) allowed affirmation of prenylation of Rap2A and identification of the preferred isoprenoid attached to Rap2A as a farnesyl-group. However, geranylgeranylation did also occur under the given conditions, but with much lower efficiency (Figure 1). The respective peak area for FPP incorporation is about 15 times the one for GGPP integration. The mutated version (C180A) was used as a negative control reaction to rule out unspecific attachment and to confirm the location of the modification.

**Figure 2:**
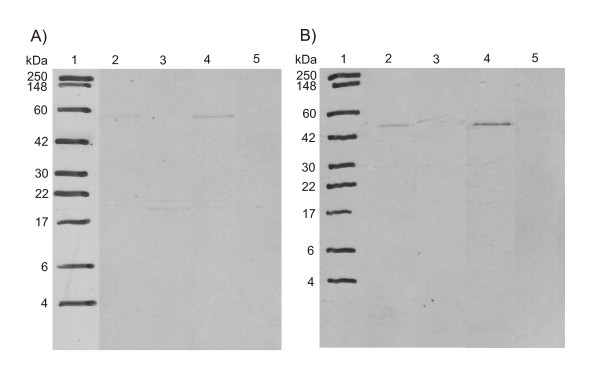
***Autoradiographs of Rap2A after exposure to radioactive prenyl anchor precursors***. Fluorography of GST-Rap2A-fusion protein on a Western membrane after treatment with En^3^Hance-spray (2-methyl-naphtalene, Perkin-Elmer), showing a protein size marker in lane 1, wildtype GST-Rap2A translated with [^3^H]mevalonic acid in lane 2, GST-Rap2A C180A with [^3^H]mevalonic acid in lane 3, GST-Rap2A with [^3^H]FPP in lane 4 and GST-Rap2A with [^3^H]GGPP in lane 5. A) film after exposure for 7 days, B) film after exposure for 20 days at -80°C. There is no sign of incorporation of GGPP as detected with the TLC-scanner, underscoring the higher sensitivity of our new method. It should be noted that it is difficult to evenly spread the En^3^Hance-substance over all membrane area. Therefore, it is not surprising that the relative signal intensities are not identical between TLC scanning and autoradiography.

All results obtained with our new method were consistent with previously reported data on Rap2A [50], demonstrating the functionality of the assay. It should be noted that the time consumption of the scanning procedure (1–2 hour per gel) is markedly reduced compared with autoradiography (weeks-months). To allow direct comparison of methods, a film has been exposed with the same Western membrane used for detection with the TLC-Scanner after application of En^3^Hance Spray from PerkinElmer for one and for three weeks at -80°C. An exposure of three weeks was necessary to get a distinct signal from all bands, which had given a strong signal with the scanner. However, it wasn't sufficient to detect the low amount of GGPP incorporated (Figure 2). If the expression of the target protein is lower than that of Rap2A, the autoradiography can require months of exposure time.

**Figure 3:**
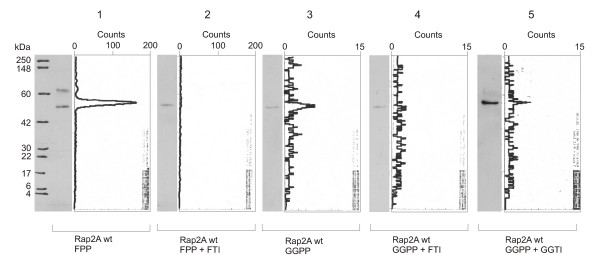
***Western blots and TLC scanning results for Rap2A incubated with prenyltransferase inhibitors***. Western Blot and corresponding scans from TLC linear analyzer of wildtype GST-Rap2A-fusion protein translated with [^3^H]FPP (lane 1), with [^3^H]FPP and 50 μM FTI-277 (lane 2), with [^3^H]GGPP (lane 3), with [^3^H]GGPP and 50 μM FTI-277 (lane 4), and with [^3^H]GGPP and 50 μM GGTI-298 (lane 5). There is no incorporation of FPP with FTI (lane 2), and there is also no incorporation of GGPP with FTI (lane 4), while a hardly detectable signal remains with GGTI (lane 5), suggesting that Rap2A is recognized only by farnesyltransferase. However, the enzyme shows some cross-reactivity with GGPP.

To determine the enzyme prenylating Rap2A, we performed the same assay with and without inhibitors of prenyltransferases. The signal yielded by incorporation of [^3^H]FPP was reduced to background level upon addition of 50 μM of the FTase inhibitor FTI-277. In addition, the already weak signal of [^3^H]GGPP-incorporation was diminished to background level by FTI, while application of a GGTase inhibitor (GGTI-298) left a small peak (Figure 3). These data suggest that Rap2A is recognized only by FTase, but the enzyme can also transfer a geranylgeranyl-group, albeit with drastically reduced efficiency (1–2 orders of magnitude) as suggested before on the basis of peptide substrate exposure to FTase [51].

### Analysis of the in vitro prenylation of RasD2, v-Ki-Ras2 and RhoA63L with the TLC scanning method

These three candidates have been selected to show the ability of our new technique to detect alternative prenylation modes. RasD2 (synonym: Rhes, BC013419) is suggested to be a farnesylation target but only due to indirect evidence [52]. Whereas K-Ras homologues such as v-Ki-Ras2 (the Q61H oncogene mutant of K-Ras4B, BC013572) are thought to be modified both by FTase and GGTase1 [53], the RhoA protein (NM_001664.2) is primarily a GGTase1 target [54].

**Figure 4:**
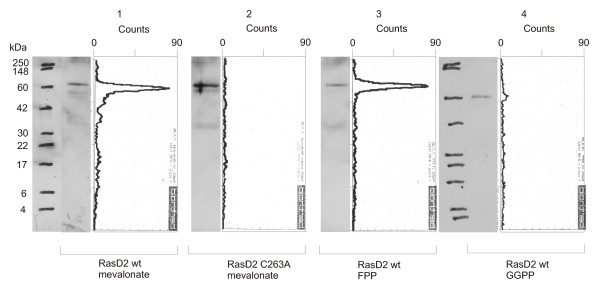
***Western blots and TLC scanning results for RasD2 with radioactive prenyl anchor precursors***. Western Blot and corresponding scans from TLC linear analyzer of wildtype GST-RasD2-fusion protein translated with [^3^H]mevalonic acid (lane 1), GST-RasD2 C263A with [^3^H]mevalonic acid (lane 2), GST-RasD2 with [^3^H]FPP (lane 3) and GST-RasD2 with  [^3^H]GGPP (lane 4). There is significant incorporation of a product of mevalonic acid (lane 1) as well as FPP (lane 3), while incorporation of GGPP is close to the detection limit (lane 4), suggesting that RasD2 is recognized primarily by the FTase.

The same *in vitro *assay was performed on all three targets. Because of lower translation efficiency, the reaction mix had to be upscaled by a factor of 5 for RasD2 and RhoA63L and a factor of 7 for K-Ras4B compared with the recipe used for Rap2A. For RasD2 and RhoA63L, we used 50 μCi of [^3^H]mevalonic acid and 25 μCi of [^3^H]FPP/[^3^H]GGPP. In the case of v-Ki-Ras2, we applied 60 and 30 μCi respectively. The results for RasD2 were similar to Rap2A with significant incorporation of a product of mevalonic acid as well as FPP, while GGPP yielded only a ca. 40 times weaker signal (as measured via area below peaks, Figure 4). Thus, we have shown with direct arguments that RasD2 is indeed a target for farnesylation [52].

**Figure 5:**
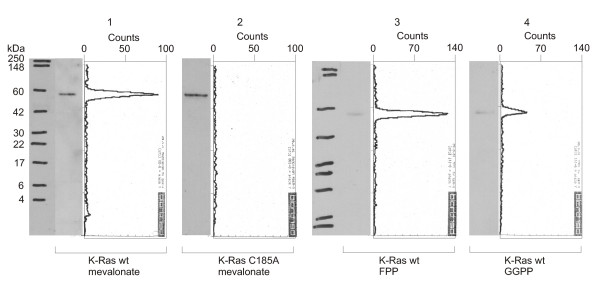
***Western blots and TLC scanning results for v-Ki-Ras2 (K-Ras-4B) with radioactive prenyl anchor precursors***. Western Blot and corresponding scans from TLC linear analyzer of wildtype GST-v-Ki-Ras2-fusion protein translated with [^3^H]mevalonic acid (lane 1), GST- v-Ki-Ras2 C185A with [^3^H]mevalonic acid (lane 2), GST-K-Ras with [^3^H]FPP (lane 3) and GST-K-Ras with [^3^H]GGPP (lane 4). There is incorporation of a product of mevalonic acid (lane 1) and FPP (lane 3) and also a reduced but noticeable amount of GGPP (lane 4), supporting the view of alternative geranylgeranylation of K-Ras in the absence of farnesylation.

**Figure 6:**
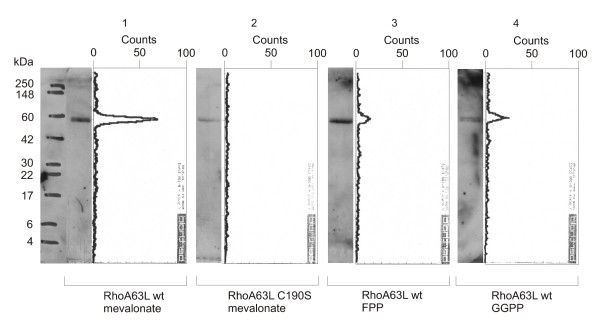
***Western blots and TLC scanning results for RhoA63L with radioactive prenyl anchor precursors***. Western Blot and corresponding scans from TLC linear analyzer of wildtype GST-RhoA63L-fusion protein translated with [^3^H]mevalonic acid (lane 1), GST-RhoA63L mutant C190S with [^3^H]mevalonic acid (lane 2), GST-RhoA63L with [^3^H]FPP (lane 3) and GST-RhoA63L with [^3^H]GGPP (lane 4). There is significant incorporation of a product of mevalonic acid (lane 1) as well as of GGPP (lane 4). The signal for FPP attachment is reduced, although more protein is detected (lane 3). This confirms GGPP as preferred substrate.

On the contrary, while also showing preference for FPP, incorporation of GGPP into v-Ki-Ras2 in the absence of FPP is only 2–3 times lower (Figure 5). These results provide strong evidence for the hypothesis of alternative prenylation while inhibiting FTase. RhoA yielded strong signals for the reactions with mevalonic acid and GGPP (Figure 6). The efficiency of FPP attachment is lower than that with GGPP by a factor of 2. Since the amount of protein detected in the Western blot under condition of FPP addition (lane 3) is considerably larger than in the case of exposition to GGPP (lane 4), we suggest that GGPP is indeed the preferred substrate. This is in accordance with the literature that RhoA is geranylgeranylated [54] and K-Ras can be modified by both isoprenoids [53].

### Electrophoretic mobility shifts of in vivo prenylated proteins

The simplest *in vivo *test for prenylation is performed with comparative electrophoretic shift analysis of non-prenylated and prenylated protein forms. The differential shift is typically not caused by the prenyl anchor attachment itself but rather by the *in vivo *post-prenylation processing steps such as subsequent palmitoylation, proteolytic cleavage of the C-terminal tripeptide of the CaaX box or C-terminal methylation. These mobility shifts are generally small and not easily detectable for all proteins due to their differential post-prenylation processing and possible variable degradation of the prenylated and non-prenylated forms.

**Figure 7:**
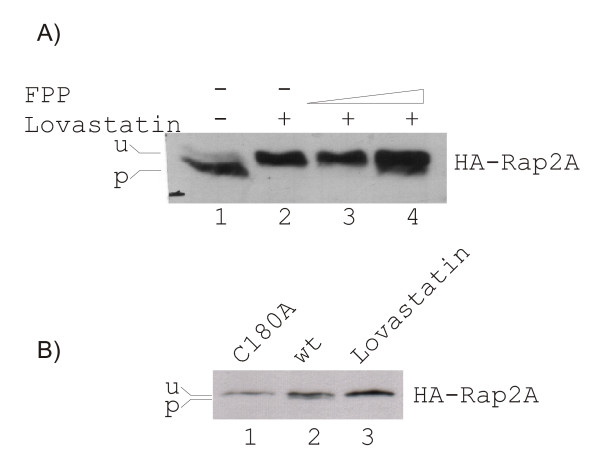
***Mobility changes of the prenylated protein form: Immunoblot analysis of Rap2A***. Western blot analysis has been performed on lysates of exponentially growing cells. U denotes the unmodified, P the prenylated form of Rap2A. A) HeLa cells have been transiently transfected with HA-Rap2A (lanes 1–4). Upon treatment with lovastatin (lane 2), the signal representing prenylated (p) Rap2A disappeared. This effect could be reversed by adding FPP (lanes 3 and 4), but not by adding GGPP (data not shown). B) HeLa cells have been transiently transfected with HA-Rap2A (lanes 2 and 3) or HA-Rap2A with a cysteine-to-alanine mutation within the C-terminal CAAX prenylation motif (lane 1, mutation C180A). The mutation and also the lovastatin treatment prevent the prenylation of HA-Rap2A.

Clear electrophoretic mobility shifts have been observed for Rap2A providing an indirect argument for its farnesylation (Figure 7). In the case of the wild-type protein, we see two bands corresponding to the non-farnesylated (slow) and farnesylated (fast) forms (lane 1). As a result of application of lovastatin (lane 2), the fast band representing the farnesylated Rap2A disappears (and the slow band grows in intensity). This effect can be reversed by application of growing amounts of exogenous FPP.

### In vivo subcellular localization of N-terminally GFP-tagged constructs

**Figure 8:**
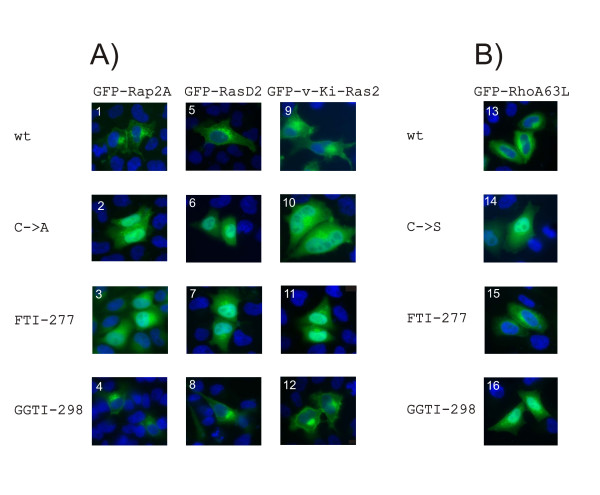
***Localization of N-terminal GFP-constructs of Rap2A, RasD2, v-Ki-Ras2 and RhoA63L in HeLa cells***. HeLa cells were analysed by fluorescence microscopy after transfection with the following constructs: inserts 1, 3 and 4 – GFP-Rap2A; insert 2 – GFP-Rap2A C180A; inserts 5, 7 and 8 – GFP-RasD2; insert 6 – GFP-RasD2 C263A; inserts 9, 11 and 12 –GFP-v-Ki-Ras2; insert 10 – GFP-v-Ki-Ras2 C185A; inserts 13, 15 and 16 – GFP-RhoA63L; insert 14 – GFP-RhoA63L C190S. Nuclei were co-stained with DAPI (blue color). A) GFP-Rap2A, GFP-RasD2 and GFP-v-Ki-Ras2 are membrane-localized with (4, 8, 12) or without (1, 5, 9) GGTI-298 treatment. Mutation of the Cys in the CaaX box (2, 6, 10) or treatment with FTI-277 (3, 7, 11) cause mislocalization and accumulation of the fusion proteins in the nucleus. B) GFP-RhoA is membrane localized with (15) or without (13) FTI-277 treatment. Mutation of the Cys in the CaaX box (14) or treatment with GGTI-298 (16) cause mislocalization and accumulation of RhoA in the nucleus.

To confirm the biological relevance of the results from our *in vitro *assays, we analyzed the subcellular localization in HeLa cells of the same proteins as N-terminal GFP-fusion constructs. We tested the wildtype forms, the variants with a mutation at the prenylation site and the wildtype forms together with FTI and GGTI (Figure 8). Fluorescence microscopic views of Rap2A and RasD2 expression showed definite membrane localization for the wildtype protein without and with GGTI. The mutant proteins and the wildtype proteins treated with FTI mislocalized and accumulated in the nucleus. A GFP-fusion protein of RhoA63L, which has been shown to be a primary geranylgeranylation target and which has been previously used for localization studies [55], was used to demonstrate the functionality of the GGTI treatment. Membrane localization is observed for wildtype protein without and with FTI, nuclear mislocalization is found for the mutant and wildtype protein with GGTI. These observations agree with the results from the *in vitro *prenylation assay.

Further, we investigated the subcellular localization of the GFP-v-Ki-Ras2 fusion protein in HeLa cells. As shown in Figure 8 (part 9), fluorescence microscopy clearly revealed that the fusion protein was co-localized with cellular membranes. A GFP fusion construct harboring a Cys to Ala mutation within the CaaX box predominantly accumulated in the nucleus (Figure 8, part 10). When using specific inhibitors of farnesylation (FTI-277) or of geranylgeranylation (GGTI-298), we surprisingly found that v-Ki-Ras2 was present mainly in the nucleus with FTI-277 (Figure 8, part 11), whereas GGTI-298 did not show any affect on the localization of the fusion protein (Figure 8, part 12).

In the literature, K-Ras4A and K-Ras4B have been reported to be predominantly farnesylated *in vivo*. In the presence of potent FTIs, both proteins were alternatively prenylated by geranylgeranyltransferase-1 in the human colon carcinoma cell line DLD-1 and COS cells [53]. Respectively, K-Ras4A and K-Ras4B were found to be associated with the membrane fraction independent of the kind of prenylation in COS cells. For complete inhibition of K-Ras4B prenylation, a combination for FTI-277 and GGTI-298 was required as examined in five different human carcinoma cell lines from pancreatic, pulmonary, and bladder origins [56]. The differing results can be due to differences in cell lines, Ras substrates or GFP-v-Ki-Ras2 overexpression. In the latter case, the ratio of prenylpyrophosphate to substrate protein is skewed. Indeed, at high expression levels, GFP-v-Ki-Ras2 was always found predominantly in the nucleus, independent of the presence of FTIs, GGTIs or the Cys-to-Ala mutation within the C-terminal CaaX box. In support of our interpretation, Rilling *et al*. [57] reported that protein prenylation in Chinese hamster ovary cells can vary as a function of the extracellular mevalonate concentration. Fortunately, only for v-Ki-Ras2, we found the localization studies to be technically tricky, fragile and the results difficult to reproduce. Whereas cells were sensitive for overexpression of wildtype GFP-v-Ki-Ras2 resulting in low transfection efficiency and, consequently, the number of transfected cells was low, no similar difficulties could be observed for GFP-vi-K-Ras2 mutant C185A or any of the other GFP-fusion constructs of RasD2, Rap2A or RhoA.

### Analysis of the in vivo prenylation of Rap2A with the TLC scanning method

**Figure 9:**
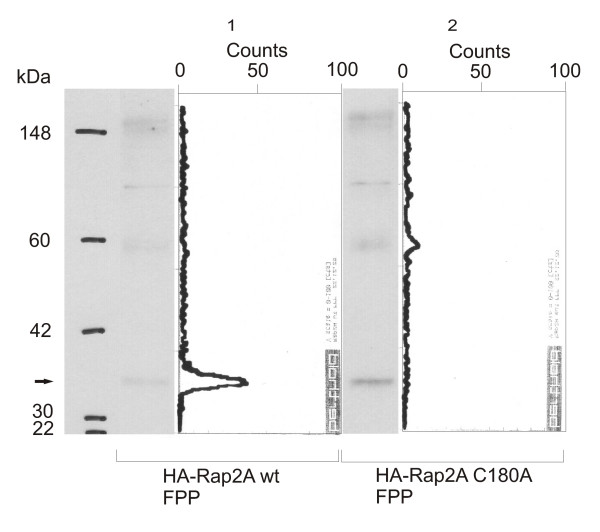
***Western blots and TLC scanning results for Rap2A with radioactive FPP in vivo***. Western Blot and corresponding scans from TLC linear analyzer of wild-type HA-Rap2A-fusion protein (lane 1) and HA-Rap2A C180A (lane 2) immunoprecipitated from HeLa-cells, treated with lovastatin after transfection with the respective plasmid construct and exposed to ^3^H-FPP. There is significant incorporation of FPP into the wild-type protein (lane 1), but no signal is detected for the C180A mutant (lane 2), demonstrating the applicability of the approach for *in vivo *labeling experiments.

It would be desirable to test whether the TLC scanning method is applicable also for the investigation of protein targets exposed to metabolic labelling with radioactive precursors *in vivo *and purified with immunoprecipitation from cell culture, SDS-page gel electrophoresis and Western transfer. Since we expected the translation efficiency to be critical for the success of the experiment, we selected Rap2A as test target (Figure 9). Indeed, it was possible to clearly show incorporation of radioactive FPP into Rap2A overexpressed in HeLa cells and recovered by immunoprecipitation with anti-HA-antibodies (lane 1) and the absence of the anchor in the C180A mutant treated identically (lane 2). It is especially notable that the amount of purified protein can be evaluated with an anti-HA-antibody on the same Western blot that was used previously for TLC-scanning similarly to the *in vitro *protocol with the anti-GST-antibody.

### Attempts to detect the prenylation status of Rap2A with HPLC-based purification methods

We have also attempted the proof of prenylation with chromatographic methods. In one of these variants, we utilized coupled *in vitro *transcription/translation and prenylation. But labelling with ^3^H-marked isoprenoids was replaced by usage of [^35^S]methionine during translation, while the added isoprenoid was not radioactively marked. Purification via GST-beads was performed in analogy to the method described above but, after precipitation with acetone, the protein was resuspended in a denaturation buffer containing 50 mM Tris-HCl pH 8.0 as well as 4 mM dithiothreitol (DTT) and 8 M urea. After denaturation, the solution was diluted by addition of 10 volumes 50 mM NH_4_HCO_3_. Following digestion with trypsin, the peptides were separated using a C18 column in reverse phase HPLC. Radioactivity was detected by scintillation counting of the collected fractions after the UV signal was recorded. Since the C-terminal peptide of Rap2A contains a methionine residue, a radioactive signal should be found at a retention time characteristic for farnesylated peptides, while it should be absent for the C180A mutant, because the non-prenylated peptide would elute much earlier. Although the expected behavior was observed in singular experiments, we have not been able to select experimental conditions for the reproducible detection of prenylated peptides (see discussion).

## Discussion

The critical step in classical prenylation assays is the detection of the radioactive anchor with autoradiography/fluorography. Unfortunately, the sensitivity of this approach is weak since the ^3^H-labeled anchors emit low-energy radiation and the amount of purified protein with attached anchor is typically very low before expression and modification conditions have been optimized individually for the specific target. The necessary exposure time is *a priori *unknown and varies widely depending on the target even after optimization of experimental conditions. Several experimenters reported long exposure times of up to several weeks (7 – 30 days [49], 3 – 14 days [48], at least one week [58]). To confirm the efficiency of our protocol, we did a comparison of our detection method to a Western membrane treated with Perkin Elmer "En^3^Hance-spray", which is the membrane equivalent to a gel soaked in "Amplify" scintillation liquid, and found weak signals after one week and distinct signals only after three weeks (Figure 2, compared to Figure 1). TLC scanning is an efficient alternative to autoradiography/fluorography.

Even having a negative outcome after 2 months exposure does not clarify whether the protein investigated cannot be prenylated or whether the amount of protein after purification is simply too low, for example due to unspecific adsorption to test tubes promoted by the prenyl anchor. It should be noted that the issue is not resolved with a [^35^S]methionine-based control of translation in a parallel experiment. However, this problem is circumvented by another advantage of the present protocol. It has the possibility to evaluate the amount of protein directly from the Western blot that has been used for TLC scanning. If a band is detected with low intensity of anti-GST- or anti-HA-antibody binding, this indicates that the expression of the target protein must be up-scaled.

Filter binding assays (i.e., the separation of proteins transcribed and translated *in vitro *in the presence of radioactive anchor precursors from free anchors with filters) provide another quick alternative to autoradiography. The information from such a test is limited since there is no size resolution of the protein mixture and no possibility to directly evaluate the amount of target protein. Furthermore, free radioactive anchors will non-specifically adsorb to the filter material or proteins resulting in high background signals.

To our knowledge, the current method is the first one utilizing a TLC-Scanner for the analysis of putative targets of PTases on Western blot membranes. Compared to autoradiography/fluorography, this approach reduces the detection time from several weeks/months to 20 minutes per lane, resulting in an overall time effort for the whole experiment of about three days, given that the cDNA of the GST- (or HA-tag-) fusion protein is already available. Additionally, this assay detects incorporated ^3^H-label and translation efficiency of the same reaction, rendering control reactions with [^35^S]methionine redundant and reducing variability of results caused by pipetting inaccuracies. In conclusion, the TLC scanning method is more sensitive and offers a more reliable way of quantification of any covalently linked ^3^H-labeled posttranslational modifications in much less time compared with autoradiography. Especially, when conditions for *in vitro *or *in vivo *protein expression and incubation still need to be set up or optimized, this method dramatically enhances chances of generating successfully generating reproducible results in reasonable time since the experimental cycle is considerably shortened.

The use of a GST-tag (or a HA-tag) provides a way of removing free radioactive label as well as separation from highly abundant proteins from the rabbit reticulocyte (*in vitro *test) or cell culture lysate (*in vivo *test), resulting in lower background signal. In addition for proteins with lower translation efficiency than Rap2A, it offers the opportunity to use bigger reaction volumes or larger cell cultures and load the whole yield on a gel without exceeding its capacity. Furthermore, it provides the opportunity to use the same primers and antibodies for all investigated proteins, making the adaptation of the assay to screening with higher throughput only a small step.

We have shown that the results obtained with our TLC scanning method are in line with those from parallel experiments testing electrophoretic mobility shifts (Figure 7) or subcellular localization changes (Figure 8) due to prenyl anchor attachment. It should be emphasized that the latter two methods are indirect and leave room for alternative interpretations whereas our assays based on TLC scanning provide a much stronger argument. It unequivocally shows anchor incorporation into the target protein directly *in vitro *as well as *in vivo*.

Unfortunately, we were not able to find any conditions, which reproducibly allowed detection of the prenylated peptide with HPLC-based purification methods after proteolytic digest. Most of the times, there was no visible difference between wildtype and mutant Rap2A in UV-signals as well as radioactivity measurements, except the fact that the total peptide content was significantly (ca. 3 times) higher for the mutant protein. These findings suggest that, in contrast to the non-prenylated protein, a considerable amount of prenylated protein is lost by unspecific adsorption to the walls of the Eppendorf tubes, vials, tubes and microwell plates used for the reaction and following processing steps. This is in agreement with our observation from the Western blots of the TLC scanning method (Figures 1, 2, 3, 4, 5, 6 and 9), which in almost all cases showed much higher protein content for the non-prenylatable mutant proteins. Additionally, we derived much better results when performing reaction and purification steps the same day, storing the samples in SDS-PAGE sample buffer at -20°C over night. Storage of the protein in the reaction mix or in PBS without detergent resulted in decreased radioactive signals. From these observations, we suggest that there would be even higher losses of the prenylated peptide after digestion, since the properties of the shorter polypeptide are much more dominated by the hydrophobic isoprenoid group, leaving only undetectable amounts of labeled prenylated peptide in solution.

These problems show that the chromatographic method might not be applicable for the small amounts of protein yielded by *in vitro *transcription/translation. There might be the possibility to overcome most of the troubles by simply increasing the amount of target protein. Results obtained with Rap2A expressed in HeLa-cells, purified by immunoprecipitation and digested with trypsin showed significant discrepancy between the UV-signals of wildtype and mutant protein (data not shown). One peak with a retention time close to the one of FPP had a peak area ~10 times larger for wildtype protein, while all other peaks were nearly identical. Based upon these promising preliminary experiments, future work may find a mass spectrometry-coupled HPLC-based approach useful for *in vivo *prenylation analysis.

We think that the mechanistic role of the prenyl anchor for the biological function of the proteins studied in this work still requires additional research. For the convenience of the reader, we summarize the current state of knowledge with respect to the molecular and cellular functions of the investigated protein targets for prenylation in the following four paragraphs. Apparently, Rap2A, RasD2, K-Ras and RhoA need the prenyl anchor to get translocated into the right signaling context by membrane association. Rap2 has been shown to promote integrin activation [59] and to directly bind to the actin cytoskeleton of platelets [60]. Rap2A is regulated by the same GEFs and GAPs as Rap1, but with much lower efficiency for the GAPs. This results in a high ratio of GTP-bound protein. Rap2 may be a slow molecular switch with functions similar to Rap1, but while the latter transduces strong, transient signals, Rap2A could determine the basal level. Thus, Rap1 would be required in the initial step of cell adhesion, which is then maintained by Rap2 signaling [61].

RasD2/Rhes (ras homolog expressed in striatum) is expressed predominately in the striatum [62] but also in thyroid glands and pancreas β-cells [63]. It is involved in selected stritial functions, mainly locomotor activity and motor coordination [64]. Unlike the Ras proteins, RasD2 does not activate the ERK-pathway, but it binds and activates phosphoinositide 3-kinase (PI3K). Additionally, RasD2 impairs activation of cAMP/PKA pathway by thyroid stimulating hormone (THS), as well as by activated β2-adrenergic receptor, suggesting a regulating function upstream of activation of the respective heterotrimeric G-protein complex. The mechanism of action implies uncoupling of the receptor from its downstream target [52].

The Ras proteins have been reported to be involved in many signaling pathways, regulation cell differentiation and proliferation as well as cell shape and motility, to mention only the most important. Ras proteins are GTPases that function as molecular switches, being active in GTP-bound state and inactive when GDP-bound. The different Ras proteins show high homology to each other and collaborate in a complex network, making it hard to distinguish whether their functions are provided by all of them or are unique for a certain type of Ras protein. Nevertheless, there is some experimental data indicating specific functions of K-Ras4B in cell-cell and cell-matrix contacts as well as in apoptosis [65]. These presumptions are supported by the fact that K-Ras4B has a different strategy for membrane association than H-Ras, N-Ras and K-Ras4A, with a polylysine stretch in the hypervariable region instead of cysteine residues as palmitoylation sites. This comes along with localization of K-Ras4B to different microenvironments of membranes and also a trafficking pathway different from the other Ras proteins [66].

In humans, there are three highly homologous isoforms of Rho GTPases, called RhoA, B and C [67]. Similar to the Ras proteins, their activities are highly overlapping, explaining why reported functions are hardly ever assigned to a certain family member. Regulation of the actin cytoskeleton, particularly the formation of stress fibers, was the first reported function of Rho. Further investigations have revealed roles in the regulation of cell polarity, gene transcription, G1 cell cycle progression, microtubule dynamics and vesicular transport pathways [68]. Thus, it seems that Rho proteins play a major role in vital cell functions such as morphogenesis, chemotaxis, axonal guidance and cell cycle progression [69].

## Conclusion

Savings in time for experimentation and the higher sensitivity for detecting ^3^H-labeled lipid anchors recommend the TLC-scanning method with GST-tagged or HA-tagged target proteins as the method of choice for analyzing their prenylation capabilities *in vitro *and *in vivo *and, possibly, also for studying the myristoyl and palmitoyl posttranslational modifications.

## Methods

### Construct production and cloning

We generated plasmids containing GST- and pEGFP-fusions of all genes studied in this work. The cDNAs of Rap2A (IMAGE clone ID IMAGp998M0310712, Genbank accession BC070031), RasD2 (IMAGp958D21250, BC013419), v-Ki-Ras2B (IMAGp998J059643Q1, BC013572) and the open reading frame coding for RhoA63L (provided as pEGFP C1-vector by C. J. Der, UNC USA; in contrast to the wild type form, this mutant is permanently activated and is able to induce malignant transformation of cells [55]) were cloned into the pGEX5X1-vector (pGEX4T1 for RhoA63L), thereby creating N-terminal GST-fusion proteins. The Stratagene QuikChange XL Site-Directed Mutagenesis Kit was used to introduce cysteine-to-alanine mutations in the CaaX-motifs. Since this residue is the site of covalent thioether linkage of the isoprenoid modification, the ability to become modified should be abolished. For RhoA63L, the already available cysteine-to-serine mutant (cloned into the pEGFP C1-vector as supplied by C. J. Der, UNC USA) has been used. N-terminal GFP-fusion proteins were used to investigate the subcellular localization in transiently transfected HeLa-cells. Therefore, both wildtype and mutant cDNA of Rap2A, RasD2, and v-Ki-Ras2 were also cloned into the pEGFP C2-vector.

### In vitro prenylation assay

The cDNA of the GST-fusion proteins was amplified by PCR using standard conditions. A 5'-primer has been designed especially for *in vitro *transcription/translation, containing a promoter, a Kozak-Consensus-sequence and an annealing sequence for the GST-tag: 5' gcgtaatacgactcactatagggagaccaccatgtcccctatacttaggttattgg 3' A 3'-primer sequence 5' agatcgtcagtcagtcacgat 3' has been designed to anneal in the pGEX5X1-vector downstream of the insert, allowing the use of the same primer pair for all proteins. All oligonucleotides used were synthesized by MWG Biotech. The radioactive label of choice (typically, 20 μCi [^3^H]mevalonic acid, 10 μCi [^3^H]FPP or [^3^H]GGPP, all purchased from American Radiolabeled Chemicals) was dried in a speedvac under vacuum at room temperature to remove the solvent, since ethanol could disrupt the transcription/translation reaction. 20 μl rabbit reticulocyte lysate, 0.5 μl PCR-Enhancer, 0.5 μl methionin (all supplied with the Promega TNT Quick Coupled Transcription/Translation Kit) and 2.5 μl of the PCR-reaction were added, mixed and incubated at 30°C for 4 hours. For experiments with inhibitors of prenyltransferases, the whole mixture including 50 μM of the appropriate inhibitor, but without the DNA, was incubated for 30 min on ice. Then, the reaction was started by addition of DNA. The following steps were identical in all experiments. During the incubation of the reaction mixture, 50 μl glutathione sepharose 4B-beads (75% slurry, from Amersham Biosciences) were separately resuspended in 0.5 ml PBS and spun down in a microcentrifuge at 1.600 rpm for one minute. The supernatant was removed and the washing step repeated once to equilibrate the beads for protein binding. The whole TNT-reaction-mix and PBS to a final volume of 200 μl was added. After resuspension, the beads were incubated with gentle agitation at room temperature for 1 hour. Afterwards, they were washed 5 times with 0.5 ml PBS. Following the last washing step, 50 μl of elution buffer (10 mM reduced glutathione in 50 mM Tris-HCl, pH 8.0) were added and incubated again for 1 hour with agitation. The beads were spun down, the supernatant transferred to a fresh vial and the protein precipitated by addition of 0.5 ml ice-cold acetone. The mixture was spun at 10.000 rpm for 1 minute. The supernatant was carefully decanted and the pellet air-dried for 10 minutes.

The pellet was re-suspended in sample buffer, incubated at 80°C for 10 min and resolved by SDS-PAGE (15%). The protein was transferred from the gel to a nitrocellulose membrane by electroblotting. The membrane was left to dry. Each lane was scanned separately for 20 min using a Berthold TLC linear analyzer LB 282. The spatial resolution of the scanner allows to assign each signal to a certain protein size. Following this measurement, the membrane was blocked with 10% milk powder in PBS. After incubation with primary antibody (anti-GST-antibody from rabbit, 1:5000) and secondary antibody (ECL Anti-rabbit IgG, Horseradish peroxidase linked whole antibody from donkey purchased from Amersham Biosciences, 1:10.000), addition of ECL plus Western Blotting Detection solution and exposure of a Hyperfilm ECL (both from Amersham Biosciences) for 15 seconds, a band at a molecular weight corresponding to the signals measured by the TLC analyzer is detectable.

### Determination of differential electrophoretic mobility after expression in cell culture

For N-terminal tagging, the ORFs of Rap2A (wild-type) and the Rap2A C180A mutant form were cloned into the plasmid pCIneo-HA [70]. HeLa cells were cultured on 6-well plates in DMEM/10% FCS to 50% confluency. We transiently transfected the cells with 1 μg DNA using Lipofectamine Reagent and Plus Reagent (Life Technologies) according to the manufacturers manual. After 3 hours of incubation, the transfection medium was replaced by fresh DMEM/10% FCS with or without 50 μM Lovastatin (Sigma). For analyzing the effect of a FPP gradient, two samples with either 2 μM or10 μM FPP have been prepared.

Ca. 16 hours later, extracts were prepared with lysis buffer (50 mM Hepes, 140 mM NaCl, 1 mM EDTA, 1% (v/v) Triton X-100, 0.1% (w/v) Sodium Deoxycholic acid, Complete Protease Inhibitor Cocktail (Roche)). Before loading the samples onto a 16% SDS Gel, the extracts were centrifuged for 10 minutes at 13000 rpm using a table top centrifuge and the supernatants boiled with sample buffer for 5 minutes. The proteins were transferred to nitrocellulose membranes and probed with mouse anti-HA 12CA5 antibodies and HRP-conjugated secondary antibodies.

### Determination of intracellular localization

HeLa cells were plated at low density on coverslips. Then, they were transfected with GFP-expression vectors carrying the cDNA's of Rap2A, RasD2, v-Ki-Ras2 and RhoA63L using Lipofectamine and Plus Reagent in serum-free medium (Life Technologies) for 3.5 h. After washing, the cells were maintained in growth medium for 14 h. Cells were rinsed with PBS, fixed in 2% formaldehyde in PBS for 20 min, washed with PBS, permeabilized with 0.1% Triton X-100 in PBS for 10 min, washed with PBS and mounted in vectashield (vector laboratories) for direct fluorescence of GFP. The effect of farnesylation or geranylgeranylation inhibitors was assessed by treatment of the cells with FTI-277 (10 μM) or GGTI-298 (5 μM) (Sigma) during maintenance in growth medium for 14 h. Cells were observed using an Axiplan 2 Imaging Microscope (Zeiss). GFP- as well as DAPI-images were acquired with a Coolsnap HQ camera (Photometrics) and analyzed using the software Metamorph 6.2r4 (Universal Imaging Corp.).

### In vivo prenylation assay with HA-tag-based immunoprecipitation from cell culture and Western blot TLC scanning

#### Transfection and labelling

For N-terminal tagging, the ORFs of Rap2Awt and Rap2AC180A were cloned into the plasmid pCIneo-HA [70]. HeLa cells were cultured on 6 well plates in DMEM/10% FCS to 50% confluency. We transiently transfected the cells with 1 μg DNA using Lipofectamine Reagent and Plus Reagent (Life Technologies) according to the manufacturers manual. After 3 hours of incubation, the transfection medium was replaced by DMEM/10% FCS/30 μM Lovastatin (Sigma). Four hours later, the medium was replaced by DMEM/10% FCS/30 μM Lovastatin (Sigma) containing 400 μCi ^3^H-FPP (ARC).

#### Immunoprecipitation

After ca. 16 hours, extracts were pre-cleared with Dynabeads M-280 Sheep anti-Mouse IgG (Dynal) in lysis buffer (50 mM Hepes, 140 mM NaCl, 1 mM EDTA, 1% (v/v) Triton X-100, 0.1% (w/v) Sodium Deoxycholic acid, Complete Protease Inhibitor Cocktail (Roche)) at room temperature for 3 hours. Immunoprecipitation was performed with mouse anti-HA 12CA5 antibodies crosslinked to magnetic Dynabeads M-280 Sheep anti-Mouse IgG (Dynal) at 4°C over night. We washed the beads 3 times with lysis buffer and twice with lysis buffer containing 500 mM NaCl. Before loading the samples onto a 10 % SDS Gel, the beads were boiled in sample buffer for 5 minutes. The proteins were transferred to nitrocellulose membranes and monitored for incorporation of ^3^H-FPP anchors by TLC analysis. Subsequently, we probed the Western blots with mouse anti-HA 12CA5 antibodies and HRP-conjugated secondary antibodies. Magnetic beads were washed three times with TBS-T (0.01% Triton X-100) and incubated with mouse anti-HA 12CA5 crude serum at 4°C over night.

#### Crosslinking to beads

The beads were again washed 3 times with TBS-T and 3 times with 0.2 M Sodiumborate pH 9.0. We crosslinked beads and antibodies with 20 mM DMP in 0.2 M Sodiumborate pH 9.0 for 15 minutes at room temperature. Finally, the beads were washed 3 times for 15 minutes with 1 M Tris pH 8.0 and 3 times with TBS-T.

## Abbreviations

FPP, farnesylpyrophosphate; FTase, farnesyltransferase; FTI, farnesyltransferase inhibitor; GAP, GTPase activating protein; GEF, guanine nucleotide exchange factor; GFP, green fluorescent protein; GGPP, geranylgeranylpyrophosphate; GGTase1, geranylgeranyltransferase 1; GGTase2, geranylgeranyltransferase 2; GGTI, geranylgeranyltransferase inhibitor; GST, glutathione-S-transferase; HA, hemagglutinin; PBS, phosphate-buffered saline; PCR, polymerase chain reaction; PTases, prenyltransferases; PTM, posttranslational modification; SDS-PAGE, sodium dodecyl sulphate polyacrylamide gelelectrophoresis; TLC, thin layer chromatography

## Authors' contributions

FE initiated this research. The experiments were performed by WB and MK. WB, MK, FP and FE conceived the work and advised modifications of experimental designs. The sequence-analytic part was carried out by SMS. WB, MK and FE contributed to the writing of the manuscript and all authors read and approved the final text.

## References

[B1] Glomset JA, Gelb MH, Farnsworth CC (1990). Prenyl proteins in eukaryotic cells: a new type of membrane anchor. Trends Biochem Sci.

[B2] Moores SL, Schaber MD, Mosser SD, Rands E, O'Hara MB, Garsky VM, Marshall MS, Pompliano DL, Gibbs JB (1991). Sequence dependence of protein isoprenylation. J Biol Chem.

[B3] Caplin BE, Hettich LA, Marshall MS (1994). Substrate characterization of the Saccharomyces cerevisiae protein farnesyltransferase and type-I protein geranylgeranyltransferase. Biochim Biophys Acta.

[B4] Zhang FL, Casey PJ (1996). Protein prenylation: molecular mechanisms and functional consequences. Annu Rev Biochem.

[B5] Maurer-Stroh S, Washietl S, Eisenhaber F (2003). Protein prenyltransferases. Genome Biol.

[B6] Maurer-Stroh S, Washietl S, Eisenhaber F (2003). Protein prenyltransferases: anchor size, pseudogenes and parasites. Biol Chem.

[B7] Maurer-Stroh S, Eisenhaber F (2005). Refinement and prediction of protein prenylation motifs. Genome Biol.

[B8] Rilling HC, Breunger E, Epstein WW, Crain PF (1990). Prenylated proteins: the structure of the isoprenoid group. Science.

[B9] Glomset JA, Farnsworth CC (1994). Role of protein modification reactions in programming interactions between ras-related GTPases and cell membranes. Annu Rev Cell Biol.

[B10] Fukada Y, Takao T, Ohguro H, Yoshizawa T, Akino T, Shimonishi Y (1990). Farnesylated gamma-subunit of photoreceptor G protein indispensable for GTP-binding. Nature.

[B11] Maltese WA, Wilson AL, Erdman RA (1996). Prenylation-dependent interaction of Rab proteins with GDP dissociation inhibitors. Biochem Soc Trans.

[B12] Kloog Y, Cox AD (2004). Prenyl-binding domains: potential targets for Ras inhibitors and anti-cancer drugs. Semin Cancer Biol.

[B13] Kuroda Y, Suzuki N, Kataoka T (1993). The effect of posttranslational modifications on the interaction of Ras2 with adenylyl cyclase. Science.

[B14] Musha T, Kawata M, Takai Y (1992). The geranylgeranyl moiety but not the methyl moiety of the smg-25A/rab3A protein is essential for the interactions with membrane and its inhibitory GDP/GTP exchange protein. J Biol Chem.

[B15] Porfiri E, Evans T, Chardin P, Hancock JF (1994). Prenylation of Ras proteins is required for efficient hSOS1-promoted guanine nucleotide exchange. J Biol Chem.

[B16] Kisselev O, Ermolaeva M, Gautam N (1995). Efficient interaction with a receptor requires a specific type of prenyl group on the G protein gamma subunit. J Biol Chem.

[B17] Hancock JF, Magee AI, Childs JE, Marshall CJ (1989). All ras proteins are polyisoprenylated but only some are palmitoylated. Cell.

[B18] Casey PJ, Solski PA, Der CJ, Buss JE (1989). p21ras is modified by a farnesyl isoprenoid. Proc Natl Acad Sci U S A.

[B19] Cox AD, Der CJ (1992). The ras/cholesterol connection: implications for ras oncogenicity. Crit Rev Oncog.

[B20] Bos JL (1989). ras oncogenes in human cancer: a review. Cancer Res.

[B21] Malumbres M, Barbacid M (2003). RAS oncogenes: the first 30 years. Nat Rev Cancer.

[B22] Hahn WC, Counter CM, Lundberg AS, Beijersbergen RL, Brooks MW, Weinberg RA (1999). Creation of human tumour cells with defined genetic elements. Nature.

[B23] Schlessinger J (2000). Cell signaling by receptor tyrosine kinases. Cell.

[B24] Gschwind A, Fischer OM, Ullrich A (2004). The discovery of receptor tyrosine kinases: targets for cancer therapy. Nat Rev Cancer.

[B25] Jaffe AB, Hall A (2002). Rho GTPases in transformation and metastasis. Adv Cancer Res.

[B26] Sahai E, Marshall CJ (2002). RHO-GTPases and cancer. Nat Rev Cancer.

[B27] Ishida D, Kometani K, Yang H, Kakugawa K, Masuda K, Iwai K, Suzuki M, Itohara S, Nakahata T, Hiai H, Kawamoto H, Hattori M, Minato N (2003). Myeloproliferative stem cell disorders by deregulated Rap1 activation in SPA-1-deficient mice. Cancer Cell.

[B28] Marshall MS, Davis LJ, Keys RD, Mosser SD, Hill WS, Scolnick EM, Gibbs JB (1991). Identification of amino acid residues required for Ras p21 target activation. Mol Cell Biol.

[B29] Hori Y, Kikuchi A, Isomura M, Katayama M, Miura Y, Fujioka H, Kaibuchi K, Takai Y (1991). Post-translational modifications of the C-terminal region of the rho protein are important for its interaction with membranes and the stimulatory and inhibitory GDP/GTP exchange proteins. Oncogene.

[B30] Der CJ, Cox AD (1991). Isoprenoid modification and plasma membrane association: critical factors for ras oncogenicity. Cancer Cells.

[B31] Kato K, Cox AD, Hisaka MM, Graham SM, Buss JE, Der CJ (1992). Isoprenoid addition to Ras protein is the critical modification for its membrane association and transforming activity. Proc Natl Acad Sci U S A.

[B32] Kohl NE, Mosser SD, deSolms SJ, Giuliani EA, Pompliano DL, Graham SL, Smith RL, Scolnick EM, Oliff A, Gibbs JB (1993). Selective inhibition of ras-dependent transformation by a farnesyltransferase inhibitor. Science.

[B33] Gibbs JB, Oliff A, Kohl NE (1994). Farnesyltransferase inhibitors: Ras research yields a potential cancer therapeutic. Cell.

[B34] Kohl NE, Omer CA, Conner MW, Anthony NJ, Davide JP, deSolms SJ, Giuliani EA, Gomez RP, Graham SL, Hamilton K, . (1995). Inhibition of farnesyltransferase induces regression of mammary and salivary carcinomas in ras transgenic mice. Nat Med.

[B35] Mazieres J, Pradines A, Favre G (2004). Perspectives on farnesyl transferase inhibitors in cancer therapy. Cancer Lett.

[B36] Doll RJ, Kirschmeier P, Bishop WR (2004). Farnesyltransferase inhibitors as anticancer agents: critical crossroads. Curr Opin Drug Discov Devel.

[B37] Glenn JS, Watson JA, Havel CM, White JM (1992). Identification of a prenylation site in delta virus large antigen. Science.

[B38] Kamiya Y, Sakurai A, Tamura S, Takahashi N (1978). Structure of rhodotorucine A, a novel lipopeptide, inducing mating tube formation in Rhodosporidium toruloides. Biochem Biophys Res Commun.

[B39] Caldwell GA, Naider F, Becker JM (1995). Fungal lipopeptide mating pheromones: a model system for the study of protein prenylation. Microbiol Rev.

[B40] Stimmel JB, Deschenes RJ, Volker C, Stock J, Clarke S (1990). Evidence for an S-farnesylcysteine methyl ester at the carboxyl terminus of the Saccharomyces cerevisiae RAS2 protein. Biochemistry.

[B41] Marcus S, Caldwell GA, Xue CB, Naider F, Becker JM (1990). Total in vitro maturation of the Saccharomyces cerevisiae a-factor lipopeptide mating pheromone. Biochem Biophys Res Commun.

[B42] Schmidt RA, Schneider CJ, Glomset JA (1984). Evidence for post-translational incorporation of a product of mevalonic acid into Swiss 3T3 cell proteins. J Biol Chem.

[B43] Schwindinger WF, Robishaw JD (2001). Heterotrimeric G-protein betagamma-dimers in growth and differentiation. Oncogene.

[B44] Lutz RJ, Trujillo MA, Denham KS, Wenger L, Sinensky M (1992). Nucleoplasmic localization of prelamin A: implications for prenylation-dependent lamin A assembly into the nuclear lamina. Proc Natl Acad Sci U S A.

[B45] Farnsworth CC, Wolda SL, Gelb MH, Glomset JA (1989). Human lamin B contains a farnesylated cysteine residue. J Biol Chem.

[B46] Eisenhaber B, Eisenhaber F, Maurer-Stroh S, Neuberger G (2004). Prediction of sequence signals for lipid post-translational modifications: insights from case studies. Proteomics.

[B47] Hancock JF (1995). Reticulocyte lysate assay for in vitro translation and posttranslational modification of Ras proteins. Methods Enzymol.

[B48] Wilson AL, Maltese WA (1995). Coupled translation/prenylation of Rab proteins in vitro. Methods Enzymol.

[B49] Wang DA, Sebti SM (2005). Palmitoylated cysteine 192 is required for RhoB tumor-suppressive and apoptotic activities. J Biol Chem.

[B50] Farrell FX, Yamamoto K, Lapetina EG (1993). Prenyl group identification of rap2 proteins: a ras superfamily member other than ras that is farnesylated. Biochem J.

[B51] Yokoyama K, Zimmerman K, Scholten J, Gelb MH (1997). Differential prenyl pyrophosphate binding to mammalian protein geranylgeranyltransferase-I and protein farnesyltransferase and its consequence on the specificity of protein prenylation. J Biol Chem.

[B52] Vargiu P, De Abajo R, Garcia-Ranea JA, Valencia A, Santisteban P, Crespo P, Bernal J (2004). The small GTP-binding protein, Rhes, regulates signal transduction from G protein-coupled receptors. Oncogene.

[B53] Whyte DB, Kirschmeier P, Hockenberry TN, Nunez-Oliva I, James L, Catino JJ, Bishop WR, Pai JK (1997). K- and N-Ras are geranylgeranylated in cells treated with farnesyl protein transferase inhibitors. J Biol Chem.

[B54] Katayama M, Kawata M, Yoshida Y, Horiuchi H, Yamamoto T, Matsuura Y, Takai Y (1991). The posttranslationally modified C-terminal structure of bovine aortic smooth muscle rhoA p21. J Biol Chem.

[B55] Solski PA, Helms W, Keely PJ, Su L, Der CJ (2002). RhoA biological activity is dependent on prenylation but independent of specific isoprenoid modification. Cell Growth Differ.

[B56] Lerner EC, Zhang TT, Knowles DB, Qian Y, Hamilton AD, Sebti SM (1997). Inhibition of the prenylation of K-Ras, but not H- or N-Ras, is highly resistant to CAAX peptidomimetics and requires both a farnesyltransferase and a geranylgeranyltransferase I inhibitor in human tumor cell lines. Oncogene.

[B57] Rilling HC, Bruenger E, Leining LM, Buss JE, Epstein WW (1993). Differential prenylation of proteins as a function of mevalonate concentration in CHO cells. Arch Biochem Biophys.

[B58] Trainin T, Shmuel M, Delmer DP (1996). In Vitro Prenylation of the Small GTPase Rac13 of Cotton. Plant Physiol.

[B59] McLeod SJ, Shum AJ, Lee RL, Takei F, Gold MR (2004). The Rap GTPases regulate integrin-mediated adhesion, cell spreading, actin polymerization, and Pyk2 tyrosine phosphorylation in B lymphocytes. J Biol Chem.

[B60] Torti M, Bertoni A, Canobbio I, Sinigaglia F, Lapetina EG, Balduini C (1999). Interaction of the low-molecular-weight GTP-binding protein rap2 with the platelet cytoskeleton is mediated by direct binding to the actin filaments. J Cell Biochem.

[B61] Ohba Y, Mochizuki N, Matsuo K, Yamashita S, Nakaya M, Hashimoto Y, Hamaguchi M, Kurata T, Nagashima K, Matsuda M (2000). Rap2 as a slowly responding molecular switch in the Rap1 signaling cascade. Mol Cell Biol.

[B62] Falk JD, Vargiu P, Foye PE, Usui H, Perez J, Danielson PE, Lerner DL, Bernal J, Sutcliffe JG (1999). Rhes: A striatal-specific Ras homolog related to Dexras1. J Neurosci Res.

[B63] Chan SL, Monks LK, Gao H, Deaville P, Morgan NG (2002). Identification of the monomeric G-protein, Rhes, as an efaroxan-regulated protein in the pancreatic beta-cell. Br J Pharmacol.

[B64] Spano D, Branchi I, Rosica A, Pirro MT, Riccio A, Mithbaokar P, Affuso A, Arra C, Campolongo P, Terracciano D, Macchia V, Bernal J, Alleva E, Di Lauro R (2004). Rhes is involved in striatal function. Mol Cell Biol.

[B65] Ehrhardt A, Ehrhardt GR, Guo X, Schrader JW (2002). Ras and relatives--job sharing and networking keep an old family together. Exp Hematol.

[B66] Ellis CA, Clark G (2000). The importance of being K-Ras. Cell Signal.

[B67] Ridley AJ (2001). Rho family proteins: coordinating cell responses. Trends Cell Biol.

[B68] Etienne-Manneville S, Hall A (2002). Rho GTPases in cell biology. Nature.

[B69] Hall A (1998). Rho GTPases and the actin cytoskeleton. Science.

[B70] Doetzlhofer A, Rotheneder H, Lagger G, Koranda M, Kurtev V, Brosch G, Wintersberger E, Seiser C (1999). Histone deacetylase 1 can repress transcription by binding to Sp1. Mol Cell Biol.

